# Cytokine Profiles in Sepsis Have Limited Relevance for Stratifying Patients in the Emergency Department: A Prospective Observational Study

**DOI:** 10.1371/journal.pone.0028870

**Published:** 2011-12-29

**Authors:** Virginie Lvovschi, Laurent Arnaud, Christophe Parizot, Yonathan Freund, Gaëlle Juillien, Pascale Ghillani-Dalbin, Mohammed Bouberima, Martin Larsen, Bruno Riou, Guy Gorochov, Pierre Hausfater

**Affiliations:** 1 Emergency Department, Hôpital Pitié-Salpêtrière, AP-HP, Paris, France; 2 Institut National de la Santé et de la Recherche Médicale (INSERM) UMR-S 945, Paris, France; 3 Department of Immunology, Hôpital Pitié-Salpêtrière, AP-HP, Paris, France; 4 Université Pierre et Marie Curie, UPMC Univ Paris 06, Paris, France; 5 Emergency Biology Laboratory, Hôpital Pitié-Salpêtrière, AP-HP, Paris, France; 6 Institut National de la Santé et de la Recherche Médicale (INSERM) UMR-S 956, Paris, France; French National Centre for Scientific Research, France

## Abstract

**Introduction:**

Morbidity, mortality and social cost of sepsis are high. Previous studies have suggested that individual cytokines levels could be used as sepsis markers. Therefore, we assessed whether the multiplex technology could identify useful cytokine profiles in Emergency Department (ED) patients.

**Methods:**

ED patients were included in a single tertiary-care center prospective study. Eligible patients were >18 years and met at least one of the following criteria: fever, suspected systemic infection, ≥2 systemic inflammatory response syndrome (SIRS) criteria, hypotension or shock. Multiplex cytokine measurements were performed on serum samples collected at inclusion. Associations between cytokine levels and sepsis were assessed using univariate and multivariate logistic regressions, principal component analysis (PCA) and agglomerative hierarchical clustering (AHC).

**Results:**

Among the 126 patients (71 men, 55 women; median age: 54 years [19–96 years]) included, 102 had SIRS (81%), 55 (44%) had severe sepsis and 10 (8%) had septic shock. Univariate analysis revealed weak associations between cytokine levels and sepsis. Multivariate analysis revealed independent association between sIL-2R (p = 0.01) and severe sepsis, as well as between sIL-2R (p = 0.04), IL-1β (p = 0.046), IL-8 (p = 0.02) and septic shock. However, neither PCA nor AHC distinguished profiles characteristic of sepsis.

**Conclusions:**

Previous non-multiparametric studies might have reached inappropriate conclusions. Indeed, well-defined clinical conditions do not translate into particular cytokine profiles. Additional and larger trials are now required to validate the limited interest of expensive multiplex cytokine profiling for staging septic patients.

## Introduction

Despite rapid improvement in health care over the past decades, sepsis continues to be a major life-threatening condition in acute care patients [1]. The rise of antibiotic resistance is also a major challenge that calls for novel biomarkers to guide and limit prescription. In the Emergency Department (ED) and intensive care unit, sepsis can be particularly difficult to distinguish from other non-infectious conditions in patients with clinical signs of acute inflammation. This issue is of outstanding importance given that treatments and outcomes greatly differ between patients with and without sepsis [2]. To date, biomarkers that are able to distinguish between systemic inflammatory response syndrome (SIRS) and the various forms of sepsis, such as severe sepsis or septic shock, are neither sensitive nor specific enough [3]–[5]. One available strategy is to monitor changes in pro- and anti-inflammatory molecules associated with the host response to pathogens. Hence, circulating levels of procalcitonin, C-reactive protein, interleukin (IL)-1, IL-6, IL-8, IL-10, tumor necrosis factor alpha (TNF-α), Fas-ligand, and monocyte chemoattractant protein 1 (MCP-1) have all been highlighted as potential markers of sepsis [6]–[16]. However, these biomarkers have mostly been studied individually and not altogether in multiparameter studies, while there is tremendous redundancy in their functions. Furthermore, in such a complex network of interaction, one cytokine can compensate for another and multiple cytokines may be involved simultaneously in a given biological function [10], [16]. We hypothesized that comprehensive profiling of serum levels of multiple cytokines would provide greater insight into their utility for staging patients with sepsis, compared with previous studies focusing on single biomarkers. Novel multiplex technologies, which rely on a combination of fluorescent-dyed microspheres associated with a two-laser flow cytometry based system, allow reliable measurement of a broad panel of cytokines using small volumes of serum [6], [14], [17]. In this study, we quantitatively analyzed 22 cytokines, chemokines and growth factors in serum samples obtained from a single-center cohort of 126 patients attending the emergency department with acute onset diseases. We have evaluated these cytokines both individually, but more importantly, together as profiles, using various multiparameter methods. The primary aim of this study was to identify cytokine profiles that would be characteristic of the various forms of sepsis, as well as the identification of potential novel therapeutic targets in sepsis.

## Methods

### Study design and patient selection

Over a four-month period, 126 patients were included in a prospective observational cohort study conducted in the Emergency department of Pitié-Salpêtrière hospital (Paris, France), an urban, 1600-bed tertiary care center and teaching hospital with 60,000 emergency department visits per year. Eligible patients met the following criteria: age over 18 years, and an acute onset medical condition defined as *at least one* of the following criteria: fever (defined by a tympanic temperature ≥38°C at the nurse triage) and/or suspected systemic infection and/or two or more SIRS criteria [18] and/or hypotension (defined as systolic blood pressure of <90 mmHg) and/or shock. We excluded patients with trauma, pregnant and breast-feeding women, patients with cardiopulmonary arrest that required basic cardiac life support measures, and those who received intravenous fluid resuscitation, antibiotics, catecholamines or intravenous corticosteroids before enrolment. Informed consent and approval by our institutional review board (Comité de Protection des Personnes Pitié-Salpêtrière, Paris, France) were obtained before onset of the study.

### Data collection

Data collected at enrollment included patient characteristics, comorbidities, vital signs, respiratory parameters, routine blood tests, suspected source of infection, microbiological culture results, patient's severity according to the Mortality in Emergency Department Sepsis score (MEDS) [19], treatments and final diagnosis. Routine biological investigations, microbiological tests and antimicrobial therapy were prescribed by ED physicians according to our ED standard of care. Included patients were classified as having SIRS, sepsis, severe sepsis, or septic shock at the time of admission by two independent ED experts physicians (V.L & G.J), according to the ACCP/SCCM criteria and to the SCCM/ESICM/ACCP/ATS/SIS International Sepsis Definitions Conference [18], [20]. In case of disagreement, the final classification was determined by a majority opinion after additional review of the patient's medical file with a third ED expert's physician (P.H). The same methodology was applied to classify patients as having viral, bacterial or infection of unknown origin (after reviewing the medical files and follow-up by the experts) in case of a diagnosis of infection not microbiologically documented. The percentage of agreement between these two independent investigators was excellent (97%) and the inter-rater reliability was high (κ = 0.93). Thirty-day mortality was recorded.

### Sample preparation and cytokine measurement

Venous blood samples were collected in the emergency room at study inclusion before any treatment (including fluid infusion) was administered, into apyrogen Becton Dickinson Vacutainer® tubes containing clot activator (Franklin Lakes, NJ, USA) and processed immediately. After centrifugation at 2000 g for 10 min, serum samples were stored at −80°C and thawed only once. Clots of fibrin were removed from defrosted samples by a second centrifugation at 12,000 g for 10 min, and these samples were immediately used for the Multiplex cytokine analysis measurement using Invitrogen Luminex® Human cytokine 25-plex antibody bead kit (Invitrogen, Carlsbad, CA, USA), which contains beads for the following cytokines: TNF-α, IL-1β, IL-1 receptor antagonist (IL-1RA), IL-2, soluble IL-2 receptor (sIL-2R), IL-4, IL-5, IL-6, IL-7, IL-8, IL-10, IL-12, IL-13, IL-15, IL-17, Chemokine ligand (CCL)11 or Eotaxin, granulocyte-macrophage colony-stimulating factor (GM-CSF), Interferon (IFN)-α, IFN-γ, CCL2 or Monocyte chimoattractant protein 1 (MCP-1), CCL3 or Macrophage inflammatory protein-1α (MIP-1α), CCL4 (MIP-1β), CCL5 or Regulated upon Activation Normal T-cell Expressed and Secreted (RANTES), chemokine (C-X-C motif) ligand 9 (CXCL9) or Monokine induced by gamma-Interferon (MIG), and CXCL10 or Interferon gamma-induced protein 10 (IP-10). The cytokines included in this analysis were chosen for several reasons: i) we used a widely available commercial panel (Invitrogen 25-plex) so that replicate studies may be easily performed; ii) This panel allows the simultaneous assessment of a large panel of both cytokines and chemokines; iii) many of them have already been involved in the pathogenesis of sepsis in previous studies; iiii) this panel includes both pro- and anti-inflammatory cytokines and thus allows assessment of a broad spectrum of immune responses.. All samples were masked for subject identity and analyzed according to the manufacturer's instructions, using a sample dilution of 1∶4. Duplicate wells containing negative controls were used to estimate background intensity. Duplicate measurements of a 7-step, 3-fold dilution series of known standards were used to fit a 5-parameters logistic curve. One patient was excluded from analysis because cytokine measurement failed for more than 33% of the 25 cytokines studied. IL-7, IP-10 and RANTES were excluded from the subsequent analyses due to inadequate bead counts or unreliable performance of standard curves. Samples with non-detectable values were replaced by zero for the purpose of continuous data analyses. All measurements were performed blinded to the clinical history.

### Statistical analysis

Quantitative data were expressed as median (minimum-maximum) values and qualitative data as numbers and percentages. The non-parametric Mann-Whitney test was used for comparison of continuous variables between the various groups of interest. Fischer's exact test or the Khi-2 test was used for comparing categorical variables. All analyses performed during this study have been performed according to a 3-step process. First, associations between individual cytokine levels and the following outcomes of interest: SIRS, sepsis, severe sepsis, septic shock, bacterial infection and bacterial infection in febrile patients were assessed in univariate analyses using the non-parametric Mann-Whitney test. Because performing several such comparisons strongly increases the statistical risk of type I error (i.e, the risk to observe false positive results), these analyses were followed by Bonferroni correction for multiple testing, when needed. Correlations between cytokine levels and severity scores were assessed using Spearman's non-parametric correlation test. Second, we built multivariate logistic regression and multiple linear regression models to identify cytokines independently associated with outcomes of interest and severity scores, respectively. This is a very important step to determine whether a given association observed in univariate analysis remains independent when other cytokines are taken into account. In these models, either the outcomes of interest or the severity scores were used as dependent variables, and all cytokines with p-values<0.20 in univariate analyses were included as explanatory variables. Third, multidimensional analyses were performed to assess whether the various conditions of interest may be distinguished based on multiple cytokine profiles. Principal component analyses (PCA) were used to visually assess whether patients with and without these outcomes of interest could be distinguished using the cytokines identified in both univariate and multivariate analyses. In PCA, each patient is represented by a single point in a 3-dimensional space in such a manner that the closer 2 patients are, the more they share similar characteristics. The main idea of these analyses is thus to assess whether patients with and without a given outcome of interest may be regrouped within two different groups or, on the contrary, cannot be distinguished. We also used agglomerative hierarchical clustering analyses, which allow delineation of subgroups of patients sharing similar characteristics within a population of interest, to assess whether patients with and without the outcomes of interest could be distinguished using the cytokines identified in both univariate and multivariate analyses. During these analyses, patients with similar characteristics are regrouped within clusters. The main idea is to check whether patients with and without a given outcome of interest are clustered within two different groups or, on the contrary, cannot be distinguished. The primary method used for agglomerating clusters was Ward's method [21]. This was performed after standardization of cytokine levels, so that each cytokine would contribute in similar manner to the final classification. Since other methods for linking clusters (single linkage, complete linkage, group average) exist, we also used these latter, and obtained similar results (data not shown). All p-values were two-tailed and statistical significance was defined as p<0.05. Statistical analyses were performed using JMP8 (SAS institute, Cary, NC) and GraphPad Prism v5.0 (GraphPad Software, San Diego, CA).

## Results

### Patients characteristics

We included 126 consecutive patients (71 men and 55 women) with a median age of 54 years (range: 19–96 years) (table 1). Among them, 99 patients had an acute infection (viral: 19, malaria: 2, tuberculosis: 3, bacterial infection: 75) and the remaining 27 patients had miscellaneous acute medical conditions (including 2 systemic vasculitis, 3 febrile neutropenia, 3 acute anaphylaxis). Documented bacterial infections were gram-negative bacilli (n = 19), gram-positive cocci (n = 12), and mycobacteria (n = 3). Primary sites of infection were pulmonary (n = 54), urinary tract (n = 16), digestive tract (n = 11), meningeal (n = 4), cutaneous (n = 3), ear-nose-throat system (n = 2), and could not be determined in 9 patients. Viral infections comprised 17 patients with respiratory tract infections along with flu-like symptoms and negative bacterial biomarkers (no viral cultures were performed), 1 patient with documented varicella-zoster infection, and 1 patient with acute viral hepatitis. One hundred and two patients had SIRS (81%), 89 (71%) had fever, 55 (44%) had severe sepsis and 10 (8%) had septic shock. Twenty-one patients (17%) were admitted in an intensive care unit. Twelve patients (9%) were deceased at 30-days follow-up. All patients with septic shock fulfilled the definition for severe sepsis, and all these latter fulfilled the definition for SIRS.

**Table 1:** Patients' characteristics.

Variable	Whole cohort (n = 126)
**Men/Women, n (%)**	71 (56%)/55(44%)
**Age, median (range), years**	54 (19–96)
**Pulse rate, median (range), beats/min**	105 (28–146)
**Respiratory rate, median (range), breaths/min**	27 (12–44)
**SBP median (range), mmHg**	126 (40–220)
**Temperature, median (range), °C**	38.2 (33.5–40.4)
**SpO_2_, median (range), %**	97 (80–100)
**GSC, median (range)**	15 (3–15)
**Creatinin, median (range), µmol/L**	71 (33–843)
**WBC, median (range), G/L**	10.2 (0.2–39.1)
**Platelets, median (range), G/L**	218 (17–805)
**Total bilirubin median (range), µmol/L**	12 (4–86)
**pH, median (range)**	7.43 (7.14–7.64)
**CRP, median (range), mg/L**	86 (3–410)
**Procalcitonin, median (range), µg/L**	0.54 (0.02–589)
**Lactate level, median (range), mmol/L**	1.8 (0.5–11.9)
**MEDS score, median (range)**	3 (0–19)
**Hospitalization, n (%)**	102 (80.9%)
**Deaths, n (%)**	12 (9.5%)

SBP: systolic blood pressure. SpO2: peripheral pulse oxymetry, GCS: Glasgow coma scale, WBC: white blood cell, CRP; C Reactive Protein, MEDS: Mortality in Emergency Department Sepsis.

### Cytokine profiles in SIRS

**Figure 1:**
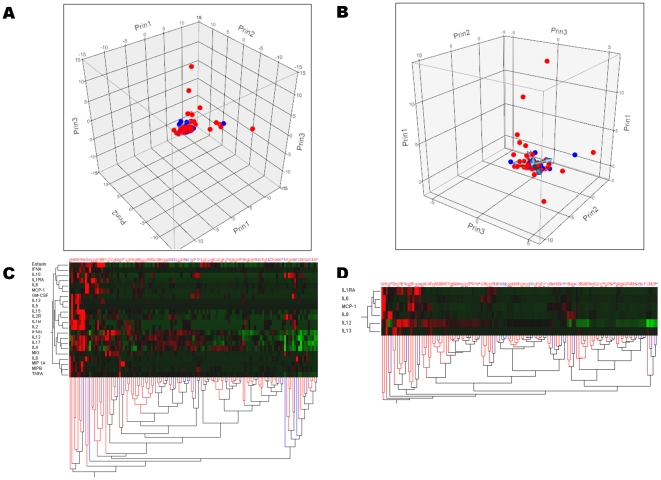
Principal component analysis and clustering of cytokine profiles in patients with (n = 102) and without (n = 24) SIRS. Patients with (red dots) and without (blue dots) SIRS are represented according to the first three components computed using principal component analysis of either all 22 cytokines (panel A), or a more limited profile using only IL1-RA, IL-6, IL-8, IL-12, IL-13 and MCP-1 (panel B). Hierarchical cluster analysis using all 22 cytokines measured (panel C) and the limited profile (panel D) are also presented. The dendrogram at the bottom of panels C & D shows the clustering of patients with (red lines) and without (blues lines) SIRS, according to the cytokine profile selected (dendrodram at the left). The color map at the center indicates the cytokine levels for each patient (brightest green is lowest level and brightest red is higher level measured). Altogether, these analyses show that SIRS patients cannot be distinguished from non-SIRS patients.

Only IL-6 serum levels were significantly increased (p = 0.02) in patients with SIRS (table 2), but this result did not reach statistical significance after Bonferroni's correction for multiple testing. Multiparameter analysis using multivariate logistic regression model revealed that no cytokine of interest was independently associated with SIRS (table 2). Both principal component analysis and hierarchical clustering analyses underlined that SIRS patients could not be segregated from the others (figure 1).

**Table 2:** Serum concentrations of cytokines in patients with and without SIRS.

	Cytokine concentration values*		P-values	
Cytokines	With SIRS (n = 102)	Without SIRS (n = 24)	Univariate†	Multivariate§
**TNF-α**	19.0 (12.5–109.6)	18.7 (11.8–24.4)	0.45	-
**IL-1β**	0.0 (0.0–244.3)	0.0 (0.0–133.8)	0.39	-
**IL-1RA**	670.7 (201.1–50,700)	513.9 (263.7–14,475)	0.17	0.37
**IL-2**	0.0 (0.0–108.5)	0.0 (0.0–85.0)	0.89	-
**sIL-2R**	828.5 (496.8–3,089)	958.1 (577.0–4,703)	0.41	-
**IL-4**	144.8 (93.2–202.7)	143.0 (78.9–213.3)	0.84	-
**IL-5**	0.0 (0.0–255.4)	0.0 (0.0–0.0)	0.49	-
**IL-6**	61.4 (0.0–11,490)	26.6 (0.0–1,637)	0.02	0.29
**IL-8**	43.4 (0.0–4,850)	19.0 (0.0–3,599)	0.09	0.59
**IL-10**	0.0 (0.0–238.9)	0.0 (0.0–302.4)	0.70	-
**IL-12**	853.0 (378.6–1,431)	834.5 (534.2–1,190)	0.12	0.40
**IL-13**	0.0 (0.0–356.7)	0.0 (0.0–0.0)	0.09	0.91
**IL-15**	0.0 (0.0–468.3)	0.0 (0.0–249.4)	0.53	-
**IL-17**	121.8 (93.3–128.2)	125.4 (95.5–177.5)	0.29	-
**IFN-α**	43.7 (31.3–155.4)	43.3 (29.0–700.0)	0.60	-
**IFN-γ**	74.0 (48.6–132.9)	72.8 (49.4–88.8)	0.65	-
**MCP-1**	454.2 (97.4–22,000)	311.0 (154.8–3,023)	0.14	0.93
**MIG**	60.1 (19.0–6,900)	63.7 (37.6–1,652)	0.92	-
**MIP-1α**	56.0 (44.7–1,749)	56.0 (49.4–680.5)	0.83	-
**MIP-1β**	83.1 (33.0–3,531)	72.9 (44.8–449.8)	0.26	-
**Eotaxin**	62.2 (21.7–462.2)	59.3 (16.8–222.6)	0.35	-
**GM-CSF**	0.0 (0.0–569.9)	0.0 (0.0–71.8)	0.29	-

*Expressed in median (min-max); unit is pg/ml. Samples with non-detectable cytokine levels were considered to be zero pg/ml.

† Assessed using the Mann-Whitney test.

§ Assessed using multiple logistic regression (all p-values<0.20 in univariate analyses were entered in the multivariate model).

### Cytokine profiles in severe sepsis

**Figure 2:**
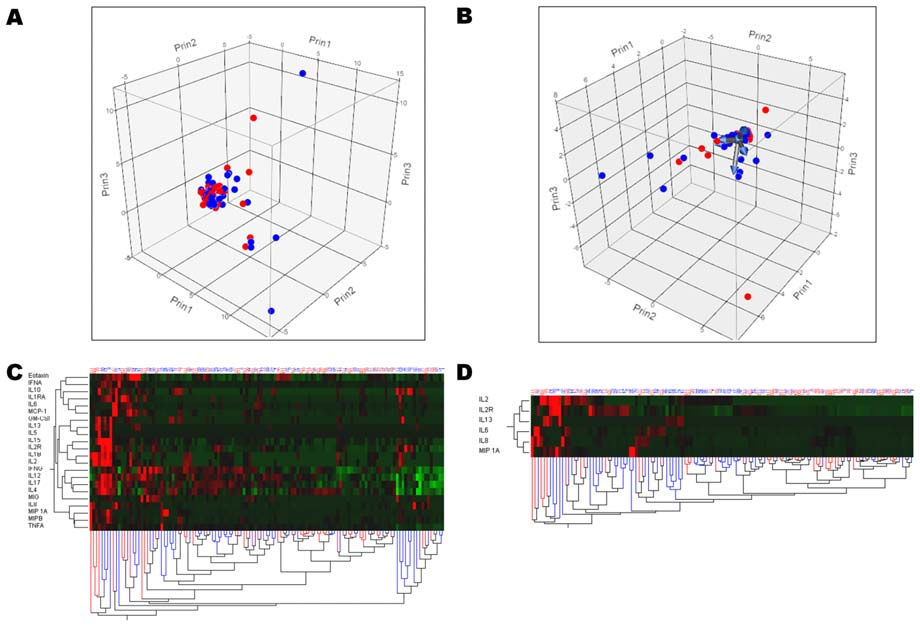
Principal component analysis and clustering of cytokine profiles in patients with (n = 55) and without (n = 71) severe sepsis. Patients with (red dots) and without (blue dots) severe sepsis are represented according to the first three components computed using principal component analysis of either all 22 cytokines (panel A), or of a limited profile using only IL-13, IL-2, sIL-2R, IL-6, IL-8, MIP-1α (panel B). Hierarchical cluster analysis using all 22 cytokines measured (panel C) and the more limited profile (panel D) are also presented. The dendrogram at the bottom of panels C & D shows the clustering of patients with (red lines) and without (blues lines) severe sepsis, according to the cytokine profile selected (dendrodram at the left). The color map at the center indicates the cytokine levels for each patient (brightest green is lowest level and brightest red is highest level measured). Altogether, these analyses show patients with severe sepsis cannot be distinguished from those without.

Serum sIL-2R was significantly decreased (p = 0.04) in patients with severe sepsis (table 3), but this result did not reach statistical significance after Bonferroni's correction. A multivariate logistic regression model revealed that sIL-2R was independently associated with severe sepsis (table 3). However, both principal component analysis and hierarchical clustering revealed that patients with severe sepsis could not be distinguished from those without severe sepsis (figure 2).

**Table 3:** Serum concentrations of cytokines in patients with and without severe sepsis.

	Cytokine concentration values*		P-values	
Cytokines	With severe sepsis (n = 55)	Without severe sepsis (n = 71)	Univariate†	Multivariate§
**TNF-α**	19.0 (14.3–109.6)	19.0 (11.8–56.3)	0.96	-
**IL-1β**	0.0 (0.0–244.3)	0.0 (0.0–220.0)	0.21	-
**IL-1RA**	749.6 (201.1–50,700)	629.1 (236.8–50,700)	0.22	-
**IL-2**	0.0 (0.0–108.5)	0.0 (0.0–90.2)	0.10	0.27
**sIL-2R**	814.7 (545.7–1,511)	866.9 (496.8–4,704)	0.04	0.01
**IL-4**	145.0 (115.7–174.3)	143.7 (78.9–213.3)	0.47	-
**IL-5**	0.0 (0.0–74.2)	0.0 (0.0–255.4)	0.85	-
**IL-6**	70.0 (0.0–11,490)	44.7 (0.0–11,490)	0.07	0.51
**IL-8**	43.5 (0.0–4,850)	31.4 (0.0–3,803)	0.20	0.94
**IL-10**	0.0 (0.0–238.9)	0.0 (0.0–302.4)	0.44	-
**IL-12**	847.4 (660.9–1,431)	851.9 (378.6–1,189)	0.95	-
**IL-13**	0.0 (0.0–96.4)	0.0 (0.0–356.7)	0.09	0.69
**IL-15**	26.5 (0.0–267.6)	27.0 (0.0–468.3)	0.75	-
**IL-17**	120.8 (97.7–145.3)	122.8 (93.3–177.5)	0.23	-
**IFN-α**	44.7 (38.0–72.3)	43.7 (29.0–699.5)	0.21	-
**IFN-γ**	73.6 (55.9–132.9)	73.6 (48.6–119.6)	0.80	-
**MCP-1**	461.0 (113.6–22,000)	383.8 (97.4–22,000)	0.42	-
**MIG**	55.8 (38.3–6,900)	63.0 (19.0–1,652)	0.25	-
**MIP-1α**	55.6 (45.9–1,749)	56.7 (44.7–680.5)	0.10	0.81
**MIP-1β**	83.5 (47.2–3,531)	80.9 (33.0–855.0)	0.80	-
**Eotaxin**	63.4 (26.1–323.5)	59.5 (16.8–422.2)	0.51	-
**GM-CSF**	0 (0.0–569.9)	0 (0.0–333.7)	0.86	-

*Expressed in median (min-max); unit is pg/ml. Samples with non-detectable cytokine levels were considered to be zero pg/ml.

† Assessed using the Mann-Whitney test.

§ Assessed using multiple logistic regression (all p-values<0.20 in univariate analyses were entered in the multivariate model).

### Cytokine profiles in septic shock

**Figure 3:**
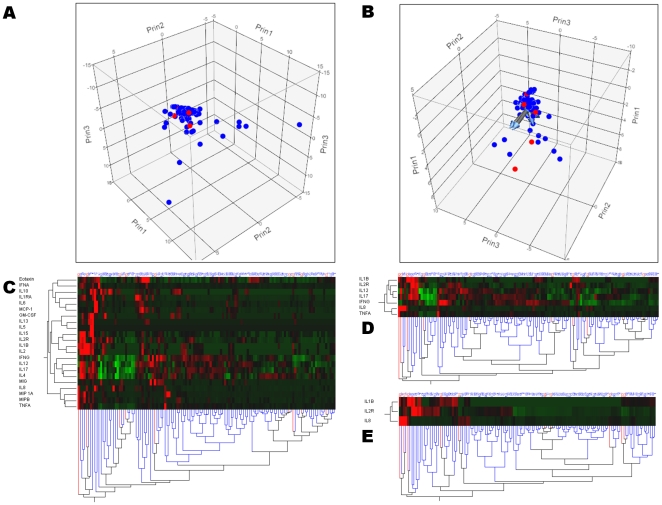
Principal component analysis and clustering of cytokine profiles in patients with (n = 10) and without (n = 116) septic shock. Patients with (red dots) and without (blue dots) septic shock are represented according to the first three components computed using principal component analysis of either all 22 cytokines (panel A), or using only IFNγ, sIL-2R, IL-1β, IL-12, IL-17, IL-8 and TNFα (panel B). Hierarchical cluster analysis using all 22 cytokines measured (panel C) and more limited profiles only based on IFNγ, sIL-2R, IL-1β, IL-12, IL-17, IL-8 and TNFα (panel D) or sIL-2R, IL-1β, and IL-8 (panel E) are also presented. The dendrogram at the bottom of panels C, D & E shows the clustering of patients with (red lines) and without (blues lines) septic shock, according to the cytokine profile selected (dendrodram at the left). The color map at the center indicates the cytokine levels for each patient (brightest green is lowest level and brightest red is highest level measured). Altogether, these analyses show patients with septic shock cannot be distinguished from those without.

IL-1β serum levels were significantly raised (p = 0.04), while IFNγ (p = 0.02) and sIL-2R (p = 0.04) were significantly decreased in patients with septic shock (table 4). However, these results did not reach statistical significance after Bonferroni's correction. Multivariate logistic regression revealed that IL-1β (p = 0.04), sIL-2R (p = 0.04) and IL-8 (p = 0.02) were independently associated with septic shock (table 4). However, both principal component analyses and hierarchical clustering analysis could not distinguish patients with septic shock from those without (figure 3).

**Table 4:** Serum concentrations of cytokines in patients with and without septic shock.

	Cytokine concentration values*		P-values	
Cytokines	With septic shock (n = 10)	Without septic shock (n = 116)	Univariate†	Multivariate§
**TNF-α**	18.0 (16.7–109.7)	19.0 (11.8–56.3)	0.19	0.93
**IL-1β**	23.5 (0.0–244.3)	0.0 (0.0–234.9)	0.04	0.046
**IL-1RA**	1,200 (236.8–50,700)	633.7 (201.0–50,700)	0.58	-
**IL-2**	0.0 (0.0–102.5)	0.0 (0.0–108.5)	0.53	-
**sIL-2R**	755.2 (545.6–1,511)	844.9 (496.8–4,703)	0.04	0.04
**IL-4**	142.8 (122.2–156.8)	144.6 (78.9–213.3)	0.41	-
**IL-5**	0.0 (0.0–0.0)	0.0 (0.0–255.4)	0.70	-
**IL-6**	126.3 (0.0–910.7)	50.8 (0.0–11,490)	0.28	-
**IL-8**	197.9 (0.0–4,850)	37.7 (0.0–3,803)	0.17	0.02
**IL-10**	0.0 (0.0–238.9)	0.0 (0.0–302.4)	0.58	-
**IL-12**	774.6 (660.9–1,431)	851.4 (378.6–1,190)	0.11	0.15
**IL-13**	0.0 (0.0–0.0)	0.0 (0.0–356.7)	0.31	-
**IL-15**	28.0 (0.0–55.8)	26.5 (0.0–468.3)	0.95	-
**IL-17**	118.8 (102.0–142.5)	122.8 (93.3–177.5)	0.19	0.79
**IFN-α**	45.0 (39.5–59.2)	43.7 (29.0–699.5)	0.97	-
**IFN-γ**	67.2 (60.0–78.4)	75.2 (48.6–132.9)	0.02	0.16
**MCP-1**	523.9 (117.9–1,723)	399.1 (97.4–22,000)	0.53	-
**MIG**	50.7 (38.3–2,952)	60.1 (19.0–6,900)	0.42	-
**MIP-1α**	56.8 (51.7–1,749)	55.8 (44.7–680.5)	0.26	-
**MIP-1β**	67.5 (51.3–3,531)	82.0 (33.0–855.0)	0.82	-
**Eotaxin**	68.6 (35.4–172.9)	61.6 (16.8–422.2)	0.85	-
**GM-CSF**	0.0 (0.0–57.0)	0.0 (0.0–569.9)	0.31	-

*Expressed in median (min-max); unit is pg/ml. Samples with non-detectable cytokine levels were considered to be zero pg/ml.

† Assessed using the Mann-Whitney test.

§ Assessed using multiple logistic regression (all p-values<0.20 in univariate analyses were entered in the multivariate model).

### Cytokine profiles in febrile patients with bacterial infection

**Figure 4:**
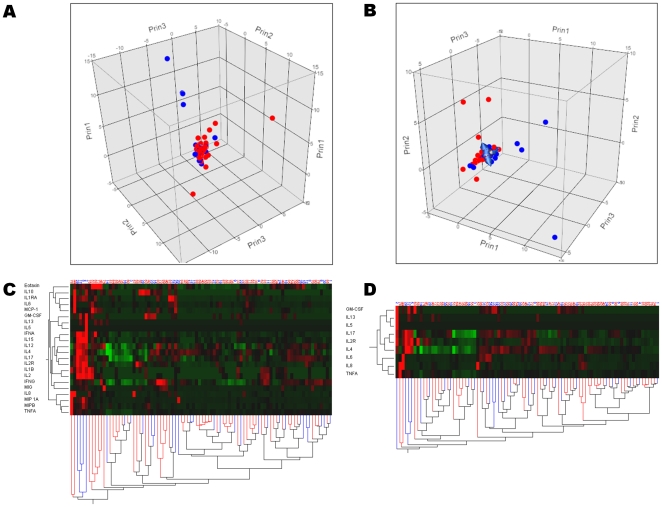
Principal component analysis and clustering of cytokine profiles in febrile patients with (n = 59) and without (n = 30) bacterial infection. Febrile patients with (red dots) and without (blue dots) bacterial infection are represented according to the first three components computed using principal component analysis of either all 22 cytokines (panel A), or a more limited profile using only GM-CSF, IL-13, IL-4, IL-5, IL-6, sIL-2R, IL-17, IL-8 and TNF-α (panel B). Hierarchical cluster analysis using all 22 cytokines measured (panel C) and the more limited profile (panel D) are also presented. The dendrogram at the bottom of panels C & D shows the clustering of febrile patients with (red lines) and without (blues lines) bacterial infection, according to the cytokine profile selected (dendrodram at the left). The color map at the center indicates the cytokine levels for each patient (brightest green is lowest level and brightest red is highest level measured). Altogether, these analyses show that febrile patients with bacterial infection cannot be distinguished from those without.

IL-8 was significantly raised (p = 0.02) and IL-17 (p = 0.01) and GM-CSF (p = 0.03) were significantly decreased in sera of febrile patients with bacterial infection compared to those without bacterial infection (table 5), but these results did not reach statistical significance after Bonferroni's correction. A multivariate logistic regression model revealed that decreased serum levels of IL-17 were independently associated with bacterial infection in febrile patients (table 5). However, both principal component analysis and hierarchical clustering analysis further demonstrated that febrile patients with bacterial infection could not be distinguished from those without bacterial infection (figure 4).

**Table 5:** Serum concentrations of cytokines in febrile patients with and without bacterial infection.

	Cytokine concentration values*		P-values	
Cytokines	Febrile patients with bacterial infection (n = 59)	Febrile patients without bacterial infection (n = 30)	Univariate†	Multivariate§
**TNF-α**	18.8 (13.2–109.7)	19.2 (12.5–25.6)	0.19	0.08
**IL-1β**	0.0 (0.0–244.3)	0.0 (0.0–220.0)	0.55	-
**IL-1RA**	666.1 (236.8–50,700)	624.4 (201.1–22,333)	0.37	-
**IL-2**	0.0 (0.0–102.5)	0.0 (0.0–90.2)	0.77	-
**sIL-2R**	817.5 (545.7–3,069)	858.7 (496.8–4,703)	0.14	0.66
**IL-4**	142.8 (96.0–173.8)	146.8 (93.2–213.3)	0.06	0.60
**IL-5**	0.0 (0.0–0.0)	0.0 (0.0–255.4)	0.16	0.99
**IL-6**	85.3 (0.0–11,490)	42.8 (0.0–2,222)	0.06	0.88
**IL-8**	47.2 (0.0–4,850)	23.1 (0.0–570.8)	0.02	0.23
**IL-10**	0.0 (0.0–238.9)	0.0 (0.0–147.8)	0.31	-
**IL-12**	850.9 (582.4–1,431)	862.7 (378.6–1,190)	0.55	-
**IL-13**	0.0 (0.0–43.1)	0.0 (0.0–356.7)	0.07	0.60
**IL-15**	27.0 (0.0–108.3)	29.0 (0.0–458.3)	0.27	-
**IL-17**	119.8 (93.3–142.5)	124.8 (97.7–177.5)	0.01	0.01
**IFN-α**	44.0 (33.3–131.7)	43.7 (38.0–155.4)	0.82	-
**IFN-γ**	72.0 (48.6–132.9)	76.0 (54.3–93.6)	0.33	-
**MCP-1**	422.5 (113.6–22,000)	395.0 (97.4–7,614)	0.55	-
**MIG**	52.9 (37.6–6,900)	61.6 (19.0–765.5)	0.59	-
**MIP-1α**	55.6 (47.1–1,749)	55.3 (44.7–642.4)	0.90	-
**MIP-1β**	84.1 (47.2–3,531)	77.5 (33.0–855.0)	0.60	-
**Eotaxin**	60.3 (28.4–422.2)	62.4 (21.7–216.8)	0.45	-
**GM-CSF**	0.0 (0.0–264.0)	0.0 (0.0–333.7)	0.03	0.17

*Expressed in median (min-max); unit is pg/ml. Samples with non-detectable cytokine levels were considered to be zero pg/ml.

† Assessed using the Mann-Whitney test.

§ Assessed using multiple logistic regression (all p-values<0.20 in univariate analyses were entered in the multivariate model).

### Cytokine profiles in patients with bacterial infection

**Figure 5:**
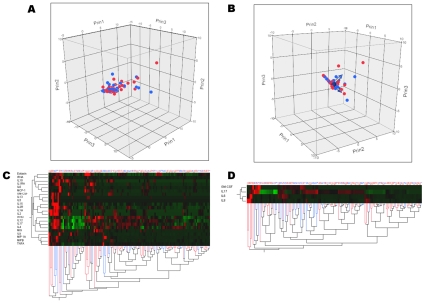
Principal component analysis and clustering of cytokine profiles in patients with (n = 75) and without (n = 51) bacterial infection. Patients with (red dots) and without (blue dots) bacterial infection are represented according to the first three components computed using principal component analysis of either all 22 cytokines (panel A), or a more limited profile using only IL-6, IL-8, IL-17 and GM-CSF (panel B). Hierarchical cluster analysis using all 22 cytokines measured (panel C) and the more limited profile (panel D) are also presented. The dendrogram at the bottom of panels C & D shows the clustering of patients with (red lines) and without (blues lines) bacterial infection, according to the cytokine profile selected (dendrodram at the left). The color map at the center indicates the cytokine levels for each patient (brightest green is lowest level and brightest red is highest level measured). Altogether, these analyses show that patients with bacterial infection cannot be distinguished from those without.

**Figure 6:**
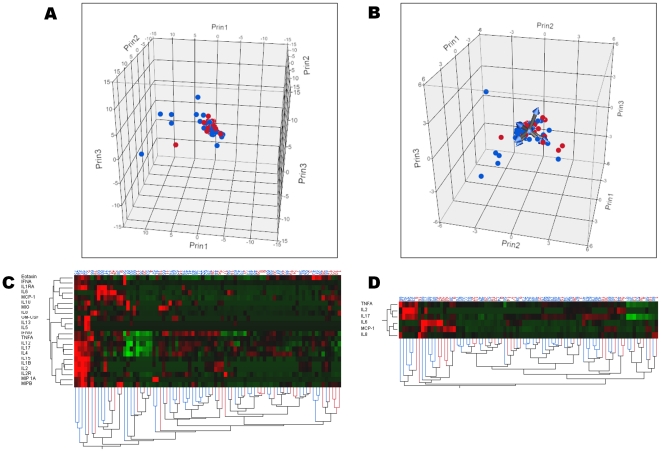
Principal component analysis and clustering of cytokine profiles in patients with proven bacterial infection (n = 31) and without (n = 51) any bacterial infection. Patients with proven bacterial infection (red dots) and without bacterial infection (blue dots) are represented according to the first three components computed using principal component analysis of either all 22 cytokines (panel A), or a more limited profile using only TNF-α, IL-2, IL-6, IL-8, IL-17 and MCP-1 (panel B). Hierarchical cluster analysis using all 22 cytokines measured (panel C) and the more limited profile (panel D) are also presented. The dendrogram at the bottom of panels C & D shows the clustering of patients with proven bacterial infection (red lines) and without bacterial infection (blue lines), according to the cytokine profile selected (dendrodram at the left). The color map at the center indicates the cytokine levels for each patient (brightest green is lowest level and brightest red is highest level measured). Altogether, these analyses show that patients with proven bacterial infection cannot be distinguished from those without bacterial infection.

IL-6 (p = 0.01) and IL-8 (p = 0.02) were significantly raised while IL-17 (p = 0.03) was significantly decreased in sera of patients with bacterial infection compared to those without bacterial infection (table 6), but these results did not reach statistical significance after Bonferroni's correction. A multivariate logistic regression model revealed that decreased serum levels of IL-17 were independently associated with bacterial infection (table 6). However, both principal component analysis and hierarchical clustering analysis further demonstrated that febrile patients with bacterial infection could not be distinguished from those without bacterial infection (figure 5). We repeated this analysis considering only the 31 patients with proven bacterial infection: IL-6 (p = 0.03), IL-8 (p = 0.02) were significantly raised in these patients compared to those without bacterial infection, while IL-17 (p = 0.02) was significantly decreased in the former (table 7). Importantly, none of these parameters remained significant after Bonferroni correction or in the multivariate analyses, and neither principal component analysis nor hierarchical clustering analysis were able to distinguish between these two groups of patients based on their cytokine profiles (figure 6).

**Table 6:** Serum concentrations of cytokines in patients with and without bacterial infection.

	Cytokine concentration values*		P-values	
Cytokines	With bacterial infection (n = 75)	Without bacterial infection (n = 51)	Univariate†	Multivariate§
**TNF-α**	18.8 (13.2–109.6)	19.0 (11.8–56.3)	0.45	-
**IL-1β**	0 (0–244.3)	0 (0–220.0)	0.60	-
**IL-1RA**	675.3 (236.8–50,700)	619.8 (201.1–22,332)	0.36	-
**IL-2**	0 (0–108.5)	0 (0–90.2)	0.64	-
**sIL-2R**	828.5 (545.6–3,069)	844.9 (496.8–4,703)	0.41	-
**IL-4**	144.1 (96.0–174.3)	145.5 (78.9–213.3)	0.46	-
**IL-5**	0 (0–74.1)	0 (0–255.4)	0.78	-
**IL-6**	85.0 (0–11,490)	34.1 (0–2,222)	0.01	0.51
**IL-8**	49.5 (0–4,850)	25.2 (0–966.9)	0.02	0.34
**IL-10**	0 (0–238.9)	0 (0–302.4)	0.29	-
**IL-12**	851.6 (582.4–1,431)	848.8 (378.6–1,190)	0.78	-
**IL-13**	0 (0–96.4)	0 (0–356.7)	0.73	-
**IL-15**	26.6 (0–267.6)	27.0 (0–468.3)	0.61	-
**IL-17**	120.8 (93.3–145.3)	124.8 (95.5–177.5)	0.03	0.04
**IFN-α**	43.7 (33.3–131.7)	43.7 (29.0–699.5)	0.64	-
**IFN-γ**	73.6 (48.6–132.9)	74.4 (49.4–93.6)	0.71	-
**MCP-1**	447.4 (113.6–22,000)	376.7 (97.4–7,613)	0.33	-
**MIG**	57.2 (37.6–6,900)	60.8 (19.0–1,652)	0.89	-
**MIP-1α**	56.2 (47.1–1,749)	55.9 (44.7–680.5)	0.92	-
**MIP-1β**	82.7 (47.2–3,531)	74.8 (33.0–855.0)	0.67	-
**Eotaxin**	61.7 (27.6–422.2)	52.9 (16.8–222.6)	0.23	-
**GM-CSF**	0 (0–569.9)	0 (0–333.7)	0.19	0.79

*Expressed in median (min-max); unit is pg/ml. Samples with non-detectable cytokine levels were considered to be zero pg/ml.

† Assessed using the Mann-Whitney test.

§ Assessed using multiple logistic regression (all p-values<0.20 in univariate analyses were entered in the multivariate model).

**Table 7:** Comparison of serum concentrations of cytokines between patients with proven bacterial infection and without bacterial infection.

	Cytokine concentration values*		P-values	
Cytokines	With proven bacterial infection (n = 31)	Without bacterial infection (n = 51)	Univariate†	Multivariate§
**TNF-α**	18.3 (13.2–29.4)	19.0 (11.8–56.3)	0.051	0.80
**IL-1β**	0 (0–86.2)	0 (0–220.0)	0.21	-
**IL-1RA**	1058.0 (263.7–7,065)	619.8 (201.1–22,332)	0.22	-
**IL-2**	0 (0–42.4)	0 (0–90.2)	0.18	0.47
**sIL-2R**	823.0 (577.0–3,069)	844.9 (496.8–4,703)	0.28	-
**IL-4**	144.1 (96.0–174.3)	145.5 (78.9–213.3)	0.73	-
**IL-5**	0 (0–74.1)	0 (0–255.4)	0.73	-
**IL-6**	96.1 (0–1,731)	34.1 (0–2,222)	0.03	0.74
**IL-8**	53.3 (0–3599)	25.2 (0–966.9)	0.02	0.38
**IL-10**	0 (0–138.7)	0 (0–302.4)	0.35	-
**IL-12**	826.6 (582.4–996.8)	848.8 (378.6–1189.7)	0.21	-
**IL-13**	0 (0–96.4)	0 (0–356.7)	0.28	-
**IL-15**	26.1 (0–267.6)	27.0 (0–468.3)	0.75	-
**IL-17**	119.8 (93.3–136.7)	124.8 (95.5–177.5)	0.02	0.23
**IFN-α**	45.0 (33.3–72.2)	43.7 (29.0–699.5)	0.36	-
**IFN-γ**	72.0 (48.6–92.8)	74.4 (49.4–93.6)	0.24	-
**MCP-1**	461.6 (189.6–3,647)	376.7 (97.4–7,613)	0.18	0.47
**MIG**	52.9 (37.6–2,951)	60.8 (19.0–1,652)	0.45	-
**MIP-1α**	55.6 (47.1–120.0)	55.9 (44.7–680.5)	0.88	-
**MIP-1β**	101.6 (51.7–516.9)	74.8 (33.0–855.0)	0.26	-
**Eotaxin**	59.4 (27.6–133.8)	52.9 (16.8–222.6)	0.97	-
**GM-CSF**	0 (0–569.9)	0 (0–333.7)	0.26	-

*Expressed in median (min-max); unit is pg/ml. Samples with non-detectable cytokine levels were considered to be zero pg/ml.

† Assessed using the Mann-Whitney test.

§ Assessed using multiple logistic regression (all p-values<0.20 in univariate analyses were entered in the multivariate model).

### Cytokine profiles and severity/mortality data

The prognostic value of serum cytokine levels was further assessed by correlating severity scores with individual cytokine levels and by studying associations between cytokine levels and mortality. Only IL-6 serum levels correlated significantly, albeit poorly, with the MEDS score (ρ = 0.27, p = 0.002). However, multivariate analysis revealed that none of the cytokines analyzed in this study was independently associated with the MEDS score. Twelve patients (9%) died during the 30-days follow-up. IL-1β (p = 0.03) and IL-12 (p = 0.03) serum levels were significantly increased in patients who died as compared to those who survived. However, multivariate analysis revealed that no cytokine was independently associated with mortality among our cohort, and that neither principal component analysis nor hierarchical clustering analysis could distinguish patients who survived from those who died (data not shown).

## Discussion

In this study, we have quantitatively analyzed 22 cytokines, chemokines and growth factors in serum samples obtained from a single-center cohort of 126 consecutive patients with acute onset diseases attending the ED of a single tertiary-care center. Unlike most studies reported in the literature, we have assessed cytokine levels not only individually, but also together as profiles using various multiparameter approaches, including multiple logistic regression, principal component analysis and hierarchical clustering. Our main finding is that previous associations commonly reported between sepsis and individual cytokine levels are not confirmed using these multiparameter techniques. Thus, we report that there are no typical cytokine profiles associated with SIRS, severe sepsis, septic shock and bacterial infection among febrile patients.

Previous studies only using univariate analyses have suggested that levels of IL-1, IL-6, IL-8, IL-10, TNF-α and MCP-1 could be raised in septic patients [4], [5], [8], [11], [22]–[25]. However, these previous studies are likely biased because: 1) univariate analyses are neither able to account for the tremendous redundancy in cytokine functions nor for the complex network of interaction that exists between them; 2) they assessed only one or two selected cytokines and thus did not take into account the higher number of pro- and anti-inflammatory cytokines that may be involved in the host response to pathogens; 3) multiple cytokine measurement with ELISA techniques is a significant source of inter-assay variability.

In the present study, we have used the recent multiplex technology which enabled us to simultaneously measure several different cytokines. This ensured that cytokine level assessment was performed homogenously. To avoid any *a priori*, we did not limit our analysis to previously reported pro- and anti-inflammatory markers of sepsis, as a wide panel of cytokines, chemokines and growth factors was assessed. These analyses revealed variations in serum cytokine levels between patients with and without SIRS, severe sepsis, septic shock and febrile patients with bacterial infection, but none of these results were significant when applying the very stringent Bonferroni's correction for multiple testing. This is an important issue, as this correction does not appear to have been used in previous studies [4], [5], [8], [11], [22]–[25].

As emphasized above, univariate analyses cannot take into account the high level of interactions taking place between the 22 cytokines studied. This limitation was overcome in the present study by further using various multiparameter analyses. We first built multivariate logistic regression models. These analyses revealed that elevated sIL-2R serum levels were independently associated with severe sepsis and, furthermore that sIL-2R but also IL-1β and IL-8, were independently associated with septic shock. Thus, IL-1β and IL-8 may be independent predictors of progression from severe sepsis to septic shock. This is further supported by two small cohort studies, consisting of 50 and 30 patients respectively, suggesting a correlation between IL-8 levels and mortality [8], [17]. We also found that IL-17 was independently associated with bacterial infection as well as with bacterial infection among febrile patients but that none of the other cytokines tested here was independently associated with SIRS. Second, we performed principal component analysis which allows processing of highly multidimensional data, using all 22 cytokine parameters studied, in addition to an analysis of more focused profiles using only cytokines of interest, as defined in univariate and multivariate analyses. These principal component analyses revealed that distinct cytokine profiles could not be attributed to patients with SIRS, severe sepsis, septic shock, bacterial infection or bacterial infection and fever. Third, we performed hierarchical cluster analyses, the results of which confirmed that patients with the various clinical outcomes included could not be regrouped and distinguished from one another on the basis of the cytokine parameters applied in this study.

While most studies reported previously were performed in intensive care units where the patients already received massive fluid resuscitation, catecholamines and/or antibiotics [5], [6], [8], [17], [23], [25], [26], the major strength of the present study is that the patient's blood samples were taken in the emergency room before they received any targeted treatment. However, we cannot formally exclude that some of the usual treatments of patients, such as statins, may have interfered with cytokine measurement or with host responses to pathogens. Because the aim of this study was to stratify *routine* patients in the Emergency Department, excluding those receiving usual treatments would have likely biased this pragmatic study. Importantly, not all patients had microbiologically documented infection, but this corresponds to the real life. It could be argued that one of the limitations of this study is the moderate cohort size, the small number of patients with septic shock (n = 10), as well as the small number of patients without inflammatory conditions. Nevertheless, the number of patients included is higher than in most previous studies and this study had sufficient power to perform robust multivariate analyses, as at least 10 patients were included for each variable in these analyses. Furthermore, the number of patients within each control group was significant as 71 patients without severe sepsis, 116 patients without septic shock and 51 patients without bacterial infection were included in the analyses. Although our analysis focused on 22 cytokines, other molecules are known to be involved, or, based on results from future studies, might be implicated in the pathogenesis of sepsis [27], [28]. We thus believe that it might be important to repeat this original analytic approach as new markers of sepsis become available. Finally, we have identified sIL-2R, IL-1β, and IL-8, but not IL-6 or TNF-α, as independent predictors of septic shock [29]–[31], while these latter two cytokines were raised in most patients. This further underlines that IL-6 & TNF-α are involved in the pathogenesis of sepsis but that they cannot be considered *independent* markers, based on our analyses.

### Conclusion

Altogether, our results suggest that previous studies aiming at identifying serum cytokines that could be relevant in sepsis could have used an inappropriate methodology. Indeed, as in previous studies, we observed that individual cytokine biomarkers appeared individually associated with severe sepsis and septic shock, but also that none of these associations remained significant upon Bonferroni's correction. Therefore, these associations should be considered exploratory until confirmed in larger prospective studies. While we have identified individual cytokines that were independently associated with severe sepsis and septic shock, or both, we have shown that unambiguous cytokine profiles corresponding to well-defined clinical groups cannot be identified in this large cohort of patients with clinical signs of acute inflammation. Therefore, our results indicate that multiplex cytokine profiling may yet be of limited interest for the pragmatic staging of septic patients in routine ED setting. Additional and larger trials are now required to validate this finding. It is, however, interesting to note that the cytokine profiles reported here are highly heterogeneous, thus suggesting that groups of patients who would be homogenous, not only clinically but also in terms of pathogens, might indeed share identical profiles. Therefore, a valuable addition for this analytic strategy could be the assessment of patients presenting highly redundant clinical patterns due to infections with well-defined pathogens [32].

### Key messages

- Previous studies aiming at identifying serum cytokines in sepsis have likely used an inappropriate methodology.
- No individual cytokine biomarker is associated with severe sepsis and septic shock.
- Cytokine profiles corresponding to SIRS, severe sepsis and septic shock cannot be identified in this large cohort of patients.
- Multiplex cytokine profiling may yet be of limited interest for the pragmatic staging of septic patients in routine ED setting.
