# Correction: Cytokine Profiles in Sepsis Have Limited Relevance for Stratifying Patients in the Emergency Department: A Prospective Observational Study

**DOI:** 10.1371/annotation/bc7e1a29-020d-4320-8f07-4dda4147fe11

**Published:** 2012-01-24

**Authors:** Virginie Lvovschi, Laurent Arnaud, Christophe Parizot, Yonathan Freund, Gaëlle Juillien, Pascale Ghillani-Dalbin, Mohammed Bouberima, Martin Larsen, Bruno Riou, Guy Gorochov, Pierre Hausfater

The equal contributions statement was omitted. Virginie Lvovschi and Laurent Arnaud contributed equally to this work. 

